# Methods for pragmatic randomized clinical trials of pain therapies: IMMPACT statement

**DOI:** 10.1097/j.pain.0000000000003249

**Published:** 2024-05-03

**Authors:** David Hohenschurz-Schmidt, Dan Cherkin, Andrew S.C. Rice, Robert H. Dworkin, Dennis C. Turk, Michael P. McDermott, Matthew J. Bair, Lynn L. DeBar, Robert R. Edwards, Scott R. Evans, John T. Farrar, Robert D. Kerns, Michael C. Rowbotham, Ajay D. Wasan, Penney Cowan, McKenzie Ferguson, Roy Freeman, Jennifer S. Gewandter, Ian Gilron, Hanna Grol-Prokopczyk, Smriti Iyengar, Cornelia Kamp, Barbara I. Karp, Bethea A. Kleykamp, John D. Loeser, Sean Mackey, Richard Malamut, Ewan McNicol, Kushang V. Patel, Kenneth Schmader, Lee Simon, Deborah J. Steiner, Christin Veasley, Jan Vollert

**Affiliations:** a Pain Research, Department of Surgery & Cancer, Faculty of Medicine, Imperial College London, United Kingdom; b Research Department, University College of Osteopathy, London, United Kingdom; c Osher Center for Integrative Health, Department of Family Medicine, University of Washington, Seattle, WA, United States; d Department of Anesthesiology and Perioperative Medicine, University of Rochester Medical Center, Rochester, NY, United States; e Anesthesiology and Pain Medicine, University of Washington, Seattle, WA, United States; f Department of Biostatistics and Computational Biology, University of Rochester, Rochester, NY, United States; g VA Center for Health Information and Communication, Regenstrief Institute, and Indiana University School of Medicine, Indianapolis, IN, United States; h Kaiser Permanente Washington Health Research Institute, Seattle, WA, United States; i Harvard Medical School, Boston, MA, United States; j Biostatistics Center and the Department of Biostatistics and Bioinformatics, Milken Institute School of Public Health, George Washington University, Rockville, MD, United States; k Department of Biostatistics, Epidemiology, and Informatics, University of Pennsylvania, Philadelphia, PA, United States; l Department of Psychiatry, Yale School of Medicine, New Haven, CT, United States; m Department of Anesthesia, University of California San Francisco School of Medicine, San Francisco, CA, United States; n Departments of Anesthesiology & Perioperative Medicine, and Psychiatry, University of Pittsburgh School of Medicine, Pittsburgh, PA, United States; o American Chronic Pain Association, Rocklin, CA, United States; p Department of Pharmacy Practice, Southern Illinois University Edwardsville, Edwardsville, IL, United States; q Department of Neurology, Harvard Medical School, Boston, MA, United States; r Department of Anesthesiology and Perioperative, University of Rochester, Rochester, NY, United States; s Departments of Anesthesiology & Perioperative Medicine, Biomedical & Molecular Sciences, Centre for Neuroscience Studies, and School of Policy Studies, Queen's University, Kingston Health Sciences Centre, Kingston, ON, Canada; t Department of Sociology, University at Buffalo, State University of New York, Buffalo, NY, United States; u Eli Lilly and Company, Indianapolis, IN, United States; v Center for Health and Technology (CHeT), Clinical Materials Services Unit (CMSU), University of Rochester Medical Center, Rochester, NY, United States; w National Institutes of Health, Bethesda, MD, United States; x University of Maryland, School of Medicine, Baltimore, MD, United States; y Departments of Neurological Surgery and Anesthesia and Pain Medicine, University of Washington, Seattle, WA, United States; z Stanford University School of Medicine, Department of Anesthesiology, Perioperative, and Pain Medicine, Neurosciences and Neurology, Palo Alto, CA, United States; aa Chief Medical Officer, MedinCell, Jacou, France; bb Department of Pharmacy Practice, Massachusetts College of Pharmacy and Health Sciences University, Boston, MA, United States; cc Department of Anesthesiology and Pain Medicine, University of Washington, Seattle, WA, United States; dd Department of Medicine-Geriatrics, Center for the Study of Aging, Duke University Medical Center, and Geriatrics Research Education and Clinical Center, Durham VA Medical Center, Durham, NC, United States; ee SDG, LLC, Cambridge, MA, United States; ff Eli Lilly and Company, Indianapolis, IN, United States; gg Chronic Pain Research Alliance, Milwaukee, WI, United States; hh Department of Clinical and Biomedical Sciences, Faculty of Health and Life Sciences, University of Exeter, Exeter, United Kingdom

**Keywords:** Clinical trial, Clinical research methods, Pragmatic trials, Comparative effectiveness research, Pain, Analgesia

## Abstract

Pragmatic, randomized, controlled trials hold the potential to directly inform clinical decision making and health policy regarding the treatment of people experiencing pain. Pragmatic trials are designed to replicate or are embedded within routine clinical care and are increasingly valued to bridge the gap between trial research and clinical practice, especially in multidimensional conditions, such as pain and in nonpharmacological intervention research. To maximize the potential of pragmatic trials in pain research, the careful consideration of each methodological decision is required. Trials aligned with routine practice pose several challenges, such as determining and enrolling appropriate study participants, deciding on the appropriate level of flexibility in treatment delivery, integrating information on concomitant treatments and adherence, and choosing comparator conditions and outcome measures. Ensuring data quality in real-world clinical settings is another challenging goal. Furthermore, current trials in the field would benefit from analysis methods that allow for a differentiated understanding of effects across patient subgroups and improved reporting of methods and context, which is required to assess the generalizability of findings. At the same time, a range of novel methodological approaches provide opportunities for enhanced efficiency and relevance of pragmatic trials to stakeholders and clinical decision making. In this study, best-practice considerations for these and other concerns in pragmatic trials of pain treatments are offered and a number of promising solutions discussed. The basis of these recommendations was an Initiative on Methods, Measurement, and Pain Assessment in Clinical Trials (IMMPACT) meeting organized by the Analgesic, Anesthetic, and Addiction Clinical Trial Translations, Innovations, Opportunities, and Networks.

## 1. Introduction

Pragmatic, randomized, controlled trials (RCTs) of pain therapies are designed to generate evidence directly relevant to clinical and health policy decision making.^48,157^ In pain research and elsewhere, these trials are increasingly valued for their potential to study intervention effects under conditions of usual care (ie, their effectiveness), especially of complex interventions.^60^ Pragmatic trials are also increasingly conducted to facilitate the translation of evidence-based interventions from highly controlled efficacy research into clinical practice.^149^ To produce results that are applicable to target populations and scenarios, these trials are frequently integrated into normal clinical practice or reproduce aspects of it.^24,172^ Relatedly, many pragmatic trials are comparative effectiveness trials, studying interventions compared with usual care or another active treatment.^71^

Aligning trials with routine care leads to heterogeneity in various domains, for example, population characteristics, treatment delivery, adherence, concomitant treatments, and data completeness. On one hand, such heterogeneity can be desirable to answer pragmatic research questions and enhance generalizability (or external validity). On the other hand, heterogeneity poses a challenge to internal validity, potentially making it difficult to ascertain a causal relationship between study treatment and outcomes.^56,77,94,187^ Indeed, some trial processes or features of clinical practice may influence outcomes in a manner that does not contribute to answering the research question. To reduce this risk, we have previously presented general suggestions for trial design, highlighting that replication of clinical practice is not an end in itself but rather a means to producing clinically relevant findings.^73^ We highlighted that each trial design decision needs to be examined regarding external *and* internal validity^73^ and in relation to the trial's intended objectives.^133^ To inform trial design and possibly judge external validity,^21,190^ the PRagmatic-Explanatory Continuum Indicator Summary 2 (PRECIS-2) is a valuable tool. It emphasizes that the level of pragmatism in trials is not a binary decision but can be determined along a spectrum, considering each specific design choice. PRagmatic-Explanatory Continuum Indicator Summary 2 considers 9 design domains on a spectrum from very explanatory to very pragmatic (or similar to usual practice in the field). This includes considerations of participant eligibility criteria, recruitment routes, settings and resources, intervention flexibility, choice of primary outcomes, and analysis methods.^111^ However, to consider each methodological choice not only regarding its likeness to routine care but also regarding its alignment with the trial objectives and potential impact on internal validity, PRECIS-2 may require supplementation with tools that examine risk of bias.^111,164,189^ Balancing the need for generalizable results with requirements for the trial results to be informative, we suggested that heterogeneity should be encouraged where demanded by the research question (ie, trial design aligned with usual care) but reduced or monitored where possible and where not required to answer the primary research question.^73^ Ultimately, pragmatic trials gain in importance by ensuring that the trial objectives are met by optimal research design.^48^

In this article, we discuss how this principle can be applied to specific aspects of pragmatic trials in pain research and to the reporting of studies. In doing so, we presume limited prior knowledge of the pragmatic trials literature and embed pain-specific considerations amongst general and current best-practice methodology. We present methodological considerations relating to ethics, stakeholder involvement and patient engagement, piloting, treatment delivery, control conditions, patient eligibility, recruitment and retention, outcomes and outcome assessment, study monitoring, analysis, and reporting. In doing so, we argue that there are many opportunities for measuring or controlling heterogeneity in a way that has little impact on the generalizability of results or that reducing a trial's alignment with clinical practice may sometimes be warranted to enhance generalizability to a particular patient population.^189^ By facilitating nuanced interpretations and enhancing trust into trial findings, certain methods may indeed promote a trial's relevance to clinical decision making. As in other fields,^3,131,137,166^ pragmatic and comparative effectiveness trials of pain require better reporting of trial methods relevant to the assessment of how findings may apply to other settings,^71^ otherwise undermining the fundamental purpose of pragmatic trials (Box 1).

## 2. Methods of manuscript development

This article is based on a preparatory systematic review^71^ and a 2-day online consensus meeting organized by the Initiative on Methods, Measurement, and Pain Assessment in Clinical Trials (IMMPACT), part of the Analgesic, Anesthetic, and Addiction Clinical Trials, Translations, Innovations, Opportunities, and Networks (ACTTION) public–private partnership with the United States Food and Drug Administration. The methodology of the review, meeting, and recommendation development are described elsewhere.^71,73^ Meeting keynote presentations and full discussion transcripts are available online (http://www.immpact.org/meetings/Immpact24/participants24.html). The meeting's objective was to discuss important considerations and provide recommendations regarding the design, implementation, and evaluation of pragmatic and comparative effectiveness clinical trials of pain treatments to inform the planning, conduct, and reporting of such studies. To enable in-depth discussion of pertinent methods, 2 articles have been prepared based on this process,^73^ this being the second.

## 3. Consensus statement of best-practice considerations

In addition to fundamental considerations of randomization, blinding, and study group design,^73^ trial designers face challenges regarding the delivery of interventions, permissiveness of concomitant and rescue treatments, and selection of patients. How practice like these features is often limited not only by concerns for internal validity or the desire for more in-depth data acquisition but also by practical constraints of trials, such as the need to recruit sufficient and representative patients.^71^ These challenges and possible solutions for pain RCTs are described below and take-home messages listed in Box 2.

### 3.1. Ethical considerations

Ethical and regulatory frameworks for clinical trials were primarily designed for tightly controlled efficacy studies. However, specific ethical considerations for pragmatic trials exist.^58,165^ In Table 1, these considerations are summarized and applied to pragmatic RCTs involving individuals in pain.

**Table 1:** Ethical considerations in pragmatic trials and in pragmatic trials for pain.

Ethical consideration in pragmatic trials	Application to pain trials and potential solutions
*Do trials require the same ethical and regulatory oversight as efficacy trials if they test ‘usual practice’ and established interventions?* This dilemma is known as the “research/treatment distinction.” Some authors purport that this distinction blurs in pragmatic trials, reducing the need for ethical oversight.^41,92^ Conversely, if routine clinical practice or aspects thereof are changed to implement the trial, ethical assessment is required.	Most pragmatic trials in pain will impose additional requirements on patients and medical personnel (such as additional outcome collection),^71^ for which ethical approval should be obtained. Targeted recruitment of potentially vulnerable people is also common in pain research,^2,71^ requiring careful ethical assessment. Ethical approval can be streamlined for all other aspects of a trial. For example, interventions may not require detailed review if they are not modified for the trial or are not directed at patients.^12^
*Is consent required in trials that test “usual practice” and established interventions that are known to be safe?* According to the principle of patient autonomy, voluntary informed consent is required for human involvement in any experimentation. If only regarding consent as necessary to inform about added risks of experimentation, consent may be waived or simplified in pragmatic trials between low-risk interventions for which there is equipoise. This is, however, controversial and defining “low risk” is challenging^108,132^; also controversial is the need for disclosure of study methodology, including randomization.^58^	Consent can be waived or simplified under certain circumstances, many of which may apply to pragmatic trials of pain research.^19,58,90,132^ Especially noninvasive nonpharmacological interventions are believed to hold little medical risk, and streamlining of consent has been called for.^41,92^ These interventions are often studied in pragmatic pain trials.^71^ Risk assessments in pragmatic pain trials should consider patient subgroups with different risk profiles, eg, depending on comorbidities or levels of health literacy.^132^ Risk/benefit assessments may have to include consideration of other undesirable effects from testing and implementing nonefficacious interventions and low-value care, such as nocebo, behavioral, and socioeconomic effects.^72^
*How can pragmatic trials promote justice and equity in trial research?* Pragmatic trials are often considered opportunities to involve people in research that are not usually participants and to conduct research relevant to minority groups. However, involvement of such groups may also require additional methods to protect potentially vulnerable participants.^132,179^ Pragmatic trials, often embedded within health systems, also risk perpetuating their existing structural injustices.^2^	Ali et al.^2^ proposed strategies for addressing injustices and inequities in pragmatic pain research. Also, see Kelsey et al.^95^ Strategies involve* Consideration of socioeconomic trial context Effective and equitable stakeholder engagement Broad criteria for participating centers Recruitment of underserved and vulnerable populations even if additional monitoring may be required Flexibility and tailoring of interventions to subgroups Accessible data collection methods Digital tools for equitable trial participation Promotion of diversity within the research team itself

The first column is largely based on Goldstein et al.^58^

* Detailed recommendations relating to these points are referred to throughout this article.

In addition, one should consider whether conducting a pragmatic trial is ethical as opposed to a more explanatory study. Considerations to respect principles of beneficence and nonmaleficence include the nature of the research question and the availability of high-quality efficacy and safety research.^73,94^ Furthermore, the aim of the IMMPACT statements on pragmatic trials is to promote trials that are optimally designed to achieve their objectives, which is a prerequisite for ethical research in light of preventing research waste.^46,141,184^

Multicenter pragmatic trials present regulatory challenges for review boards.^135^ Conversely, lack of standardization of ethics procedures across institutions can be a barrier to conducting such trials.^32^ In pain research, establishing large research networks can help standardize and centralize procedures, but challenges still remain.^1^

### 3.2. Patient and other partner engagement

Research questions for pragmatic trials are often considered “stakeholder driven”^159^ or “important for real-world decision making.”^111^ As a result, early stakeholder engagement is crucial^94^ and may include patient partners, healthcare practitioners, policy makers, and others. Identifying and involving relevant partners holds the promise that the right problems are addressed in a manner that is efficient and translatable to clinical practice or healthcare policy.^74^ As such, people with lived experience and other partners confer different kinds of benefit at different stages of trial research. Reimbursement of lay partners can reduce barriers to participation and is ethical because it promotes equitable participation in research.^134,160^ Helpful frameworks are available to guide public engagement throughout the research cycle,^78^ notably the Patient-Centered Outcomes Research Institute (PCORI) Engagement Rubric^160^ and the UK Standards for Public Involvement.^134^

In the field of pain research, large institutional networks for the conduct of multicenter pragmatic trials have been established through extensive partner engagement with a focus on developing personal relationships. For example, the Pain Management Collaboratory (PMC^3^), a joint project between 3 US government agencies, employs inwards-facing and outwards-facing mechanisms to optimize communication with key stakeholders (eg, archiving of relevant information internally, and an outwards-facing website for stakeholders and the public). The PMC^3^ has a board of stakeholders affiliated with key government and nongovernment entities and a “patient resource group” able to support the Collaboratory as a whole as well as individual trials.^1^ Further insights can be found in Bastian et al.,^7^ and IMMPACT guidance for stakeholder engagement in pain trials is being developed.^74^

### 3.3. Piloting

Piloting and feasibility testing are essential to evaluate the feasibility of many of the below considerations in the context of individual trials, and specific considerations for pragmatic trials have been published.^16^

### 3.4. Study treatment

Pain management is rarely standardized in clinical practice,^163^ and a biopsychosocial approach to pain calls for treatment individualization and multimodal care.^80,130^ Pragmatic trials represent both an opportunity and a challenge in this context: An opportunity as they allow for the study of interventions as delivered in clinical practice and irrespective of understanding mechanisms^114^; a challenge because some oversight over the content of study treatment(s) facilitates the scientific interpretation of trials and assessment of generalizability.^77^ Furthermore, pragmatic trial designs lend themselves to the study not only of comparative effectiveness but also to the investigation of treatment combinations, interactions, and tailoring strategies (such as predictive enrolment, augmentation, or switching, although modifications of the common two-arm parallel design may be required^73^). However, it should be emphasized that only pragmatic trials with substantial size and statistical power have the potential to make any inferences regarding different treatment interactions and delivery strategies.

Considerations underpinning the study of a particular therapy in a pragmatic trial need to be made explicit and the supporting evidence base reported clearly.^191^ Such evidence should include supportive efficacy and/or mechanistic research for the treatment or core components of a treatment package. Finally, intervention design for pragmatic trials needs to be based on a good understanding of how the intervention is applied in routine clinical practice. This can be obtained, eg, through practice surveys, and development ideally involves practitioner and other stakeholder engagement.

#### 3.4.1. Treatment standardization and fidelity monitoring

Although uncommon in real-world clinical pain management, authors have repeatedly called for some standardization (or protocolization) of study treatments in pragmatic trials, monitoring of fidelity, and detailed reporting of intervention content.^94,97,191^ Especially in trials of nonpharmacological interventions, provider expertise and style of practice require consideration, balancing the need for a representative range of skillsets with acceptable levels of heterogeneity in treatment delivery. Furthermore, clinical practice may not always follow evidence-based guidance so that provider training may be required, introducing elements of standardization into a trial. The extent to which treatment flexibility needs to be controlled depends on the research question and the specifics of the intervention; opportunities to train and oversee clinicians may also be limited in trials that take place in real-world settings.^94^ Notably, dosage safety and therapeutic ranges are important to consider in drug trials, while the potential to introduce undesired heterogeneity through practitioners and intervention complexity is more pertinent in nonpharmacological therapies. However, any standardization of treatment delivery, for example, through trial-specific training and protocolization, ought to be well justified. If training and standardization exceed what is seen in routine practice of a given intervention, this needs to be clearly identified and implications for a trial's generalizability discussed. Defining core intervention components and nonpermissible modalities as a minimal step is advisable to promote fidelity and clearly differentiate trial interventions from one another.^97^

Depending on the research question, fidelity monitoring may be preferable to protocolization (also see “patient adherence” paragraph below). Documenting how the treatment is delivered during the trial can be done through a variety of, ideally low-burden, methods. Methods include observing treatment delivery in a subset of treatment sessions, reviewing treatment documentation, or collecting patient-reported data on treatment adherence. High-quality fidelity (and adherence, see below) data may allow for sensitivity analyses to develop hypotheses about subgroups, mechanisms, intervention tailoring, or implementation challenges. Further considerations regarding treatment fidelity in pragmatic pain trials of nonpharmacological interventions are published.^97^

#### 3.4.2. Titration and taper phases

Titrating or tapering are issues specific to drug studies and potentially studies of devices such as neurostimulation. Titration and taper phases are expected to be more flexible in pragmatic trials than in efficacy-focused RCTs, especially if 2 drugs are compared (as opposed to comparing several titration schedules or dosages of the same drug) or if flexible titration or tapering is required by the research question. However, unknown variations in dosage may undermine the interpretability of the results of pragmatic trials, so that adherence should be monitored where possible.

#### 3.4.3. Treatment duration

The duration of the treatment phase and often the length of follow-up are based on the characteristics of the pain, the treatment being evaluated, and the research question. Considerations are also intimately linked to how interventions are usually delivered in clinical practice, which outcomes measures are employed, and what patients expect from the intervention (considerations that likely benefit from patient involvement). In trials with people living with persistent or recurring pain, realistic interventions may range from intensive one-off treatment courses to continuous (“maintenance”) care but with similar follow-up periods; or, it may be found that time to relief or time to discontinuation are meaningful outcome measure (discussed below, outcomes section), which would require trials with variable treatment phase length.

#### 3.4.4. Patient adherence

We recommend promoting adherence in line with best practice (not *normal* practice) in the respective therapeutic field to increase the informativeness of study results. This contrasts with PRECIS-2 ratings, which disincentivize encouragement or formal adherence requirements, but we consider this an opportunity to promote trial internal validity with little interference with most pragmatic research questions. However, if acceptability and adherence levels in normal practice are part of the research question, adherence should not be promoted beyond normal practice.

As with intervention fidelity, treatment adherence should be monitored in all cases to facilitate interpretation and allow for corresponding sensitivity analyses. Its retrospective assessment or patient self-report of adherence are easily implemented and commonly used in pain trials.^97^ However, ease of use needs to be weighed against the need for high-quality adherence data. In some circumstances, technological tools could be developed, which help to corroborate patient self-report, for example, user data from apps or activity tracking through wearables,^100^ but this may not always be feasible, and there are concerns about validity, methodological standardization, meaningfulness, data protection, and bias.^9,15,84,119^

If participant adherence is disregarded, important information about the treatment's real-world acceptability is lost. Collecting qualitative information on reasons for nonadherence may supplement such assessments. Furthermore, patient adherence could figure as a secondary study endpoint (discussed below, outcomes section).

### 3.5. Comparator and control conditions

#### 3.5.1. Control conditions in comparative effectiveness designs

When comparing 2 or more active treatments, a basic level of efficacy and safety of the treatments under investigation is expected as part of comparative effectiveness trials.^79,167^ In trials without a control condition (such as no treatment, placebo, or treatment as usual); however, this claim is only inferential and needs to be acknowledged as a limiting factor. Evidence to suggest that the compared treatments are efficacious and safe should be presented along with the level of recommendation (eg, as per GRADE^64^). It has been suggested that uncontrolled trials should be avoided when the underlying efficacy research for the comparator is poor and particularly in the context of noninferiority and equivalence designs.^47^ However, current practice does not reflect this recommendation.^71^

Even in pragmatic superiority trials where the comparator is an established analgesic or complex therapy, a further control group may be beneficial: In community practice, many medications are not titrated to effective dosages, and misdiagnoses can occur,^26^ and treatment adherence varies.^128,142^ Many nonpharmacological interventions for pain yield inconsistent results^150^ and are applied variably and by therapists from a multitude of backgrounds.^49,169^ Falsely negative trials of efficacious analgesic drugs are common,^45^ and this can be expected for nonpharmacological therapies too. Without a no-treatment, placebo, or treatment-as-usual control group, it cannot be concluded that the study treatments were better than any of those. If trial results showed no difference between the groups, such a control group would demonstrate that the trial was able to distinguish an effective treatment from a less effective one, thus strengthening the conclusions drawn. We acknowledge that the addition of a further trial arm holds practical and economic challenges for the overall trial but recommend that investigators carefully consider whether this is nonetheless required so that the trial does not result in uninterpretable findings. If no control group is implemented, this decision should be justified in the trial protocol or report.

#### 3.5.2. Treatment as usual/usual care

Approximately 25% of contemporary self-declared pragmatic or comparative effectiveness trials of pain therapies employ treatment or care as usual (TAU) as comparator, occasionally adding extra components such as advice or educational sessions. Approximately 70% of those trials provide information on the potential content of TAU, and most of those also collect data on the interventions actually received by patients in this group.^71^

“Usual care” is considered the “comparator of choice” by many pragmatic trial methodologists, although operationalizing this in a trials, and particularly pragmatic trials, is fraught with challenges.^188^ Sometimes called “standard care” (there is no consensus on terminology and interpretations differ^132^), what constitutes TAU differs widely, even within the same country, and may change—sometimes rapidly,^162,188^ as last seen with the widespread adoption of telehealth during the Covid-19 pandemic.^54,115^ Furthermore, clinical recommendations and actual usual practice may differ widely so that TAU cannot be inferred from the consultation of clinical guidelines.^59,105,158,171,182,186^ It is therefore paramount to describe in detail what the TAU condition entailed in the context of each trial,^162^ which may warrant monitoring of the interventions actually received. Pascoe et al.^139^ proposed 6 aspects of TAU, which should be reported in effectiveness trials for back pain, which we modified and endorse for pragmatic analgesia trials more broadly:

(1) Who provided care (include profession, training, and experience)
(2) If and what type of self-management education or advice were provided (and in which format)
(3) Whether physical activity and movement were addressed and how
(4) If and what medications were used
(5) The dosage of any interventions received
(6) Whether care was consistent with current guidelines

This information will help readers judge the generalizability of a given trial's comparison to other contexts. For example, if “usual care” in their own context involves considerably more care than in the trial, the relative benefit of the treatment may be diminished and vice versa.

Because of their potential to introduce undue heterogeneity, we will further discuss concomitant treatments below and similar considerations apply to variability detected in the TAU arm.

#### 3.5.3. Waiting list controls

In waiting list conditions, patients expect treatment at a later time point (as opposed to true no-treatment controls, where they are informed that they will not receive treatment as part of the study). Waitlist conditions can control for the natural course of disease and regression to the mean, although not for placebo effects. However, waiting for treatment may contribute to symptom persistence in anticipation of future treatment.^50,113^ The feasibility and credibility of waitlist conditions (ie, resemblance or proximity to usual practice) depend on the healthcare system. In the United Kingdom, eg, long waiting periods are currently normal in the National Health System (NHS), especially for nonurgent referrals, such as for persistent pain,^144,168^ and may be used as a realistic control condition. Nonetheless, waiting list controls are uncommon in self-declared pragmatic RCTs of pain treatments.^71^

#### 3.5.4. No-treatment controls

In no-treatment control arms, patients are informed that they will not receive treatment as part of the trial. Therefore, patients are only monitored and prohibited the use of other commonly applied treatments.^8,145^ No-treatment groups are rare in current pragmatic trials of pain treatments^71^ possibly due to the preponderance of usual care–related comparative effectiveness questions. However, they may be relevant if the study population does not usually receive any care, as may be the case in prevalent painful conditions such as headaches.

It has been argued that open assignment to no treatment may frustrate patients and negatively impact the self-reporting of symptoms, trial retention, or the patients' condition by way of the “frustrebo” effect,^143^ although empirical evidence for this phenomenon is lacking. Frustration effects may be mitigated through patient engagement and input into the research design process, as evidence supports that many patients with pain are altruistically motivated to participate in clinical trials.^43^ Instead of no-treatment controls, approximately 15% of pragmatic pain trials opt for supposedly ineffective educational or attention control arms.^71^ Also used is blinding to the existence of interventions (and a no intervention arm) in cluster randomized trials to test new patient management options.^17^ Further trial designs can address the challenge: For example, incorporating elements of patient preference (such as partially randomized preference trials^116^), by first offering patients to participate in a randomized trial or in an observational study and using the observational cohort as additional control group, or by supplementing or emulating RCTs with registry-derived observational data.^86,118^ However, all these designs come with considerable statistical challenges that are beyond the scope of this article.

All inactive control groups have the advantage to act as internal controls that test the trial's ability to detect a difference between study treatments. There are many circumstances under which this may be warranted, including unclear evidence regarding expected effect sizes or effectiveness in the specific trial context. Advantages and disadvantages of treatment as usual vs waitlist vs true no-treatment controls likely differ depending on the trial intervention and disease population but should be considered carefully. Careful communication of trial objectives and methods during the consent process may be able to attenuate expectation effects that can differ meaningfully among these different approaches.

### 3.6. Concomitant and rescue pain treatments

In pragmatic trials of pain interventions, concomitant treatments may play an important role, but their permissibility and use is poorly reported.^71^ On one hand, allowing patients to continue taking their usual medications or seeing other providers of nonpharmacological care is reflective of normal practice and thus important for the generalizability of results.^181^ Prohibiting concomitant therapies may also disincentivize recruitment and undermine patient autonomy. On the other hand, concomitant therapy use may undermine trial interpretability if unknown to the research team.

Rescue analgesia, defined as intermittent medication intake for insufficient pain relief during a trial, may similarly confound trial results, although reflective of clinical practice. Confounding can occur when study groups use rescue medication differentially (eg, when one treatment is less effective),^30,181^ through interactions with the study drug, or by producing rescue-related adverse events. The choice of rescue medication also matters, with highly effective drugs reducing effect sizes and ineffective drugs considered unethical regarding the principles of beneficence, informed consent, and patient autonomy. Notably, concomitant and rescue treatments may also consist of nonpharmacological approaches or, even more subtle and difficult to quantify, behavioral changes such as altered self-management strategies.

All these considerations should inform trialists in the design of pragmatic trials and monitoring of concomitant and rescue analgesia. Given the risk for confounding effects, parameters of permissibility and monitoring processes of actual treatment use should be established before conducting a pragmatic trial and implemented across participating centers. If the trial objective is to replicate clinical practice, one should generally not limit the use of concomitant therapies unless there is a strong rationale to do so and considering the potential effect on generalizability. However, the use of concomitant and rescue treatments should always be carefully documented and possible implications for treatment effects considered.^30,181^ Proper adjustment for such effects, when warranted, requires the use of sophisticated causal inference methods^42^ (also see section on subgroup analyses below). To date, concomitant and rescue treatment use are rarely considered in the analysis of trials, and “*poorly described procedures and incomplete reporting are likely to hinder the interpretation, critical appraisal, and replication of trial results*” (p.3),^63^ also in pragmatic trials of pain.^71,138^ Alternatively, the use of rescue analgesia can be considered as a study outcome (Table 2).

**Table 2:** Concomitant treatments and rescue medication in pragmatic analgesia trials—design and reporting considerations.

Was rescue medication permitted (yes/no)? For what reason?
Brand and generic name(s), formulation, and administration, if possible
Allowed doses and frequency
Consequences of exceeding allowed dosage (withdrawal, treatment failure)
Specific procedures (eg, dosage or timing in relation to pain symptoms)
Delivery (prescription, over the counter)
Assessor of consumption (patient: self-report, investigator: pill count)
Used as outcome? (if so, primary, secondary, or exploratory)
Report of rescue consumption in each treatment arm
Discussion of whether rescue medication influenced trial results (if so, how?)
The same considerations apply and need to be adapted to non-drug therapies and pain self-management strategies used as concomitant or rescue treatment. In addition, also document:
Profession and qualification of treatment provider
Therapy-specific detail (eg, techniques and approaches)

List based on Grøvle et al.^63^

Obtaining reliable data on rescue and concomitant therapy use is challenging. Possible approaches to data collection include monitoring of electronic health records, billing and insurance data, and patient or physician report. However, using health records is unlikely to capture fluctuating patterns of medication use such as when used as rescue medication. Collection of these data must also be feasible and has to be validated and possibly modified for the purpose of individual trials. When reporting a pragmatic trial and having access to feasible data collection methods, we propose to consider the items shown in Table 2 and collect what is realistically possible. Finally, trialists may wish to allow and monitor concomitant analgesic therapies during a baseline period or pilot testing, then align trial procedures with real-world usage patterns.

Similar to medications, permissible and prohibited nonpharmacological concomitant therapies ought to be prespecified and their use documented, including the type of therapy, its providers, intensity, duration, and frequency. Behavioral change may have to be assessed as a potential effect mediator at follow-up,^94^ depending on the study question focusing on physical activity levels, various pain coping behaviors, and psychological health, ideally using accepted questionnaires.

### 3.7. Patient populations and study sites

#### 3.7.1. Eligibility criteria

Eligibility criteria in pragmatic trials are typically broad, reflecting routine diagnostic procedures for a specific medical issue.^111^ In the pain field, this will involve triaging for serious pathology and application of standard diagnostic procedures. Frequently in pragmatic analgesia trials, eligibility criteria refer to pain duration and localization only, but common comorbidities or medications impacted participant eligibility in a large proportion of recently reviewed trials (25% and 12%, respectively).^71^ A better approach is to focus on self-reported pain intensity or interference ratings. Furthermore, the ascertainment of underlying pain-generating diagnoses (such as rheumatological disorders or neuropathies) as per ICD diagnostic codes can be considered where possible, and always employing additional screening procedures to ensure that eligibility criteria are met, as implemented by the pragmatic trials of a large US consortium.^96^

Depending on trial objectives, the diagnosis of the underlying disease may be warranted to ascertain eligibility. If the diagnostic standard involves more sophisticated technology than what is commonly available, this is acceptable for pragmatic trials but may limit generalizability to other settings or healthcare systems. The same consideration applies to “prognostic recruitment,” where patients are eligible based on prognostic profiles (such as chronification risk^25^) (see Ref. 73). If applicable, authors should discuss if the employed diagnostic technology is expected to become more widely available and otherwise justify the decision to conduct a pragmatic trial for their research question.

Trial designers may be tempted to reduce heterogeneity by applying eligibility criteria beyond ascertainment of diagnoses or simple stratification methods, but these should be kept similarly broad, based on clinical reality, and justified considering the pragmatic question under investigation. For example, a trial could focus on children, adolescents and adults, older adults and elderly, or a single sex. Further specification is only warranted if reflective of a well-delineated clinical population for which a clinically relevant and population-specific question needs to be answered. In these cases, pragmatic trials can be highly selective, eg, only including persons of a specific underserved population within clearly defined geographic, age-related, socioeconomic, and cultural boundaries; and authors must discuss the clinical or policy relevance of their selection and implications for generalizability.

If the research question demands a broad clinical sample of patients, we propose the following pain-related domains for consideration. As major eligibility criteria, these would be more inclusive than what is usually found in efficacy trials, and indeed pragmatic trials are opportunities to conduct research for people from diverse or disadvantaged communities and with co-occurring medical or mental health conditions.^2,94^ Recruiting a representative sample may, in many cases, mean specifically targeting members of certain populations and reducing barriers to trial participation, for example, through early patient partner involvement.^95^

Rather than offering generic advice on desirable eligibility criteria, however, we encourage researchers to consider what is warranted depending on their individual research objectives. Apart from necessary diagnostic criteria, we recommend:

(1) Consider including pain of any intensity, not just ≥ 4/10, balancing clinical relevance with risk for flooring effects and the ability to detect meaningful changes. Chronic daily mild pain can be burdensome, and few clinical trials have examined treatments for this common clinical condition. Indeed, acetaminophen/paracetamol, NSAIDs, and their combination are widely available as over-the-counter medications, but little is known about their efficacy and effectiveness when used to self-treat mild pain. In addition, enrolling patients with mild pain intensity but impaired function or mood may provide adequate assay sensitivity if pain intensity is not the primary outcome, but an adverse consequence of such pain is (eg, quality of life, function, satisfaction, etc).
(2) Consider including any pain duration that fulfills diagnostic criteria.
(3) Consider not excluding patients for previous treatment nonresponse unless aligned with the pragmatic research question.
(4) Consider not excluding medical or psychiatric comorbidities and substance use disorder unless for safety reasons.

While broader eligibility criteria increase trial heterogeneity, they also enable subgroup analyses, which may be able to answer clinically relevant questions. In line with applicable CONSORT reporting recommendations (item 3), authors should discuss “…*the degree to which* [the eligibility criteria] *include typical participants and/or, where applicable, typical providers (eg, nurses), institutions (eg, hospitals), communities (or localities eg, towns) and settings of care (eg, different healthcare financing systems)*.”^191^ We extend this recommendation to encourage statistical subgroup assessment where informative.

Finally, pragmatic trials are almost absent for several prevalent pain conditions, including headaches, neuropathic pain, fibromyalgia, and injury-related pain.^71^ We encourage trials with pragmatic objectives for pertinent research questions in these groups of patients.

#### 3.7.2. Patient identification and outcome assessment through electronic health records

Pragmatic trials methods assume that patients are ideally recruited from existing patient populations of participating trial centers.^111^ However, researchers need to identify the population ultimately targeted in clinical practice and promote recruitment for practical reasons. To facilitate this, trialists may have to employ more active recruitment methods.^71^ Under some circumstances, identifying eligible patients through records may facilitate enrollment, especially if an electronic health record (EHR) system is available.^38,88^ In pragmatic trials, such identification of potential trial participants from EHRs holds the promise of overcoming limitations for generalizability inherent in recruiting exclusively through physician referral and patient self-referral, including by providing a denominator for the number of potentially eligible patients within a healthcare system. However, EHRs come with various challenges, some of which are specific to pain research, and probably limit the usefulness of EHRs for most current pragmatic trials. People in chronic pain, eg, may be coded with various diagnoses, lack a precipitating and identifiable event and are thus captured only through elaborate algorithms or natural language processing tools, which require careful testing,^22,99,170^ and always the use of additional screening procedures. In less severe conditions for which patients may rely on self-management or over-the-counter medication, these filters may not be feasible. Although a rapidly developing technology, establishing EHR systems is costly and laborious,^104^ data quality can be poor and interoperability challenging, and data protection needs careful consideration.^20^ If feasible, we encourage the use of EHRs to support enrollment. We currently warn against the overreliance on such real-world data for purposes integral to trial integrity, specifically outcome assessment,^88^ unless the EHR system used has been shown to be reliable, data quality is high, and its use is ethically justifiable.

#### 3.7.3. Recruitment and retention

According to PRECIS-2, recruitment through patients' usual appointments and from multiple diverse clinics is most pragmatic.^111^ However, insufficient recruitment strategies pose a risk to the interpretation of trial results, as they may lead to underpowered trials and the omission of relevant patient subgroups.^136^ The latter is of particular concern in chronic pain, which is more prevalent in populations that are arguably more difficult to recruit into research trials or have limited access to health care in general, such as various underserved populations, ethnic minorities, those with psychiatric comorbidities, and older adults.^13,65,66,91,146,161^ Provided these are potential or intended end users of the study treatment, maximizing generalizability may require targeted recruitment methods, and trial results could be used to advocate for improved access to effective care for people from the studied populations. Indeed, pragmatic trials of pain therapies obtained the lowest average rating for pragmatism in the recruitment domain,^71^ likely reflecting the above practical and scientific concerns.^73^

Incentives are a compelling way of enhancing recruitment, including financial compensation or free access to otherwise self-paid care. Because self-payment may be a barrier to trial participation and may cause differential attrition in the potentially less desirable trial arm, compensating patients for treatment costs is appropriate.^175^ Nevertheless, incentives may bias recruitment and could attract “professional” patients. Furthermore, trial designers ought to consider the vulnerability of patients, both financial and personal, which may create ethical dilemmas by juxtaposing equitable access to research with principles of patient autonomy, research integrity, and nonmaleficence.^136^ In the field of chronic pain in particular, the desire to find effective treatment options may be strong.^18,89^

It is unclear whether the risk of participant attrition is increased in pragmatic trials due to their longer duration (with dropout rates ranging from 0% to 63%, mean 15%, in a recent sample^71^) or lower because of reduced research burden compared with more explanatory trials.^136^ Either way, retention of participants is an important factor in the planning phase of pragmatic trials and must be considered proactively to minimize the impact of missing data. The use of imputation methods for missing data can only address the issue retrospectively and relies on untestable assumptions.^11^ Recruiting more participants to compensate for anticipated attrition is not only costly but also does not eliminate the potential bias due to missing data. Instead, stakeholder involvement and feasibility testing can help anticipate and mitigate potential retention problems,^129^ as various factors such as the treatment and research burden, associated costs, and the prohibition of concomitant or rescue treatments may interfere with successful retention. Especially in community-based pragmatic trials, creative and patient-centered approaches may be warranted, including such that build relationships and allow for flexible responses to patient needs.^55,176^ During the consent process and thereafter, it is crucial to emphasize the importance of staying in the trial once enrolled, as this can help improve retention and the validity of results.^27^

#### 3.7.4. Sample size

Good pragmatic trials provide a power calculation, ensuring adequate statistical power for the detection of all important differences to be examined.^31^ CONSORT highlights that “*[if] calculated using the smallest difference considered important by the target decision maker audience (the minimally important difference) then [authors should] report where this difference was obtained.*” (item 7).^191^ Effect sizes obtained in efficacy trials with more controlled circumstances may not be transferable to pragmatic trials. When faced with uncertainty concerning the appropriate choice of effect size for sample size determination in a pragmatic trial, it is prudent to be conservative to reduce the chance of failing to detect small yet clinically important treatment effects. Adaptive methods that adjust the required sample size based on emerging data (as discussed in Ref. 73) can be helpful in saving resources in this context. Given the potentially larger heterogeneity in pragmatic trials and different nature of pragmatic research questions, larger sample sizes may be required and should be supported by funders.^53,136^

#### 3.7.5. Study sites

Most self-declared pragmatic trials in pain research are multicenter trials.^71^ In principle, trial sites should include those that would deliver the intervention in normal practice.^111^ Irrespectively, basic quality requirements for sites apply, such as the ability to recruit and retain participants, ensuring the welfare and rights of patients, the willingness of staff to attend research-related training, and the ability to conduct and record research to current standards.^183,185^ To ensure this and especially because pragmatic questions in pain research likely require the inclusion of nonacademic centers^71^ where most care is delivered, more training and feasibility testing may be required.^183^ Trial designers should ensure that such training does not impact generalizability by changing care delivery.^111^ To promote internal validity while not reducing the generalizability of findings from common nonacademic pain care settings, trial designers may also consider the use of cluster randomization to prevent contamination, creative adherence and fidelity monitoring strategies, and assessor blinding.^51,57^ Heterogeneity between trial centers may be warranted to enhance generalizability, but its extent should be carefully assessed and documented, and the effects on trial results formally examined. At the very least, authors should comply with the respective CONSORT extension item (no. 21) and “*[d]escribe key aspects of the setting which determined the trial results.*” They should also “*[d]iscuss possible differences in other settings where clinical traditions, health service organisation, staffing, or resources may vary from those of the trial*.”^191^

Assuming that patient populations are heterogenous across and within study sites, the number of patients recruited per site might need to be considerably larger than in most efficacy trials.^136^ Overall numbers may have to be even higher in cluster-randomized trials. Here, opting for fewer patients per cluster and more clusters is preferable, rather than vice versa, while lower recruitment numbers are more compatible with individual randomization.^67,106^

### 3.8. Outcome domains and measures

Starting with a minimal set of meaningful core outcomes, trial designers ought to consider how each additional measure may alter the clinical workflow.^180^ In doing so, we recommend attention to what constitutes a feasible and realistic outcome tool, always ensuring that outcome measures are validated, reliable, and responsive to change.^29^

Commonly, outcomes in pragmatic trials should be “*of obvious importance from the patient's perspective*” and “*relevant to the people who decide whether to implement the intervention on the basis of its [the trial's] results*.” (p. 9).^111^ Ascertaining this may require early engagement of various stakeholders.^180^ In analgesia trials, the 4 core domains of pain outcomes—intensity, interference, function and change—are well established^29,173^ and relevant to pragmatic trials in the field because of their importance to patients.^174^ Several outcome measures cover these domains and are relatively quick to administer: This includes numeric rating scales (NRS) for each domain or composite score-generating measures such as the Brief Pain Inventory (BPI), the three-item PEG-scale,^102,103^ or subscales of the Patient-Reported Outcomes Measurement Information System (PROMIS) or the Short-Form 36 (SF-36).^103^ Other ultra-brief options for outcome assessment include simple clinical questions. These appear meaningful and conform with practical restraints of research in primary care settings but may require further validation for usage in trials. Examples include “Is your pain tolerable?,” allowing for simple Yes/No answers or adapted to Likert-type ratings where required.^117^ Individualization, where each patient determines which outcome is most meaningful to them, is another practically relevant option for subjective experiences such as pain.^5,83,107,154^ The addition of other outcomes relevant to particular patient populations, such as physical function, mood, sleep, adverse effects, individualized outcomes, or disease-specific measures, should be evaluated. Depending on the research objective, outcomes in pain trials are likely long term, and target effect sizes should be chosen to be clinically meaningful.

In contrast to other research areas, objectifiable endpoints that would facilitate a low-effort data collection are sparse in pain research.^178^ However, their development appears particularly relevant in the context of pragmatic trials to mitigate some of the challenges of unblinded studies,^180^ and some approaches from, eg, psychiatry or rheumatology appear relevant to pain research. Indeed, authors in the field have made attempts to implement more objective and yet potentially clinically meaningful outcome measures in trials. Promising examples include the use of remission criteria,^6,127,151^ changes in medication^101,177^ or other healthcare utilization,^25,62^ and the discontinuation of or adherence to treatment as indicators of therapeutic success.^61,123,124,126,155^ In the latter, mechanisms need to be in place to distinguish treatment failure from the occurrence of adverse events and symptom resolution.^124^ Such outcomes can be coupled with smart and adaptive trial designs, including rerandomization in the case of treatment failure,^73,76,151,152,155^ working towards personalized care. Whether the use of dichotomous outcomes over continuous variables as primary outcomes affects the trial's ability to detect an effect needs to be considered (also in light of clinical meaningfulness),^125^ although the results for both approaches should ideally be reported.

Economic considerations may impact or alter clinical decision making and health policy. Regardless, economic considerations were reported infrequently in recent self-declared pragmatic trials of pain treatments,^71^ likely because they are themselves expensive and may not be supported by funders. However, especially in comparative effectiveness trials where cost-savings of one over the other therapy are not apparent, we encourage data collection and formal cost-effectiveness analyses.

### 3.9. Assessment intensity and frequency and follow-up duration

The highest PRECIS-2 score is given when frequency and extent of follow-up assessments are limited to what would be seen in routine practice, collecting outcome data that simulates or makes use of clinical practice as much as possible.^111^ While self-declared pragmatic trials in pain tend to employ more laborious follow-up assessments than what would be expected in routine practice,^71^ we agree that excessive data collection may interfere with successful patient retention, resulting in missing data that is not necessarily reflective of normal clinical practice. Intense follow-up will also increase research burden and trial cost. Nonetheless, some standardization of symptom reports collected as part of routine practice may be required to ensure data integrity.^20^ To reduce bias and research burden, outcome data are obtained with minimal personal contact between participants and clinicians and research staff, which may require further deviation from routine practice but is feasible with current technology.^85^ However, data completeness and quality require special attention during digital outcome collection, with solutions including the continuous monitoring of data and proactive support of trial participants identified as not completing questionnaires.^4^ At the same time, while contemporary data collection and digital trial delivery methods may be options to broaden access to research, accessibility must be ensured for populations with limited technology access or literacy.^2,95^

The duration of follow-up periods tends to be longer in pragmatic trials than in explanatory trials^111^ (over 1 year on average in pain trials^71^). Follow-up periods should reflect the time horizon of patient and clinical decision making, drawing on stakeholder engagement. The need for data completeness poses challenges over potentially long periods. Either, pragmatism may have to be sacrificed to protect internal validity (eg, scheduling additional data collection calls or appointments) or follow-up periods shortened. Ideally, however, electronic data collection methods and low-burden outcome measures are employed to mitigate this risk, and preplanned strategies for handling missing data are in place (see below).

Overall, trial designers should consider carefully which data are needed to answer the research question, and how data collection and follow-up duration may interfere with trial feasibility and internal validity.

### 3.10. Study monitoring

Commonly, pragmatic trials employ centralised and on-site study monitoring to ensure participant welfare, compliance with regulatory standards, and scientific integrity.^10^ Especially in long-term and multicenter studies, adequate data monitoring enables researchers to detect and correct problems with the collection and relay of information. In trials with safety considerations, the implications are even more profound, but the timely collection of safety data while minimizing follow-up visits represents a major challenge of pragmatic trials.^82^

Risk-based or risk-proportionate approaches to monitoring are employed in many pragmatic trials.^10^ Either, monitoring processes are streamlined according to an initial risk assessment (eg, of the potential for harm from different interventions) or in-depth evaluations are triggered based on prespecified indicators of risk to participant safety, data integrity, or trial conduct.^10^ We advocate for such approaches, as they minimize interference of monitoring processes with clinical practice and promote the feasibility of large trials.^82,121^

### 3.11. Analysis

Analysis methods are an important area where pragmatic trials can become more efficient and ensure that their results are as informative as possible. As for all clinical trials, prospective registration is essential for pragmatic trials. This should include a detailed statistical analysis plan to prevent selective reporting. Objectives should be prioritized and accompanied by statistical formulations.^28^

#### 3.11.1. Estimands and missing data

The most common analysis in pragmatic trials includes all randomized patients irrespective of treatment adherence, performing what is often called an “intention-to-treat” (ITT) analysis.^71,111^ However, such analysis often requires imputing missing data and thus necessitates careful consideration of assumptions underlying the handling of missing data. For example, making up missing data with the Last Observation Carried Forward method assumes that participants continue their symptom trajectory after dropping out of the trial, while Missing at Random or Baseline Observation Carried Forward imputations have very different assumptions.^33^ Furthermore, ITT analyses may not always correspond to the most relevant clinical questions, for which one may want to take into account patients' levels of treatment adherence, reasons for patient noncompliance, or the effects of using nonprotocol therapies.

For the handling of missing data and other events that may interfere with the interpretation of trial results, the field should move towards the use of estimand frameworks.^34^ Estimands are comprehensive frameworks defining how missing data and disruptive (“intercurrent”) events are handled to provide a clearer and more accurate picture of the real-world impact of treatments. In a first step, the clinical question to be answered must be carefully specified. From this, the estimand can then be formulated, the components of which are the treatment conditions to be studied, the target population, the outcome variable of interest, and the population summary that will be used as the basis for the treatment comparison. An important consideration for defining the estimand is how so-called intercurrent events will be handled, including discontinuation of treatment (and reason), use of rescue treatment, noncompliance, use of prohibited treatments, or other protocol violations. Once the estimand has been established, the principles underlying its construction should guide strategies for dealing with missing data. Because established statistical methods for accommodating missing data depend on assumptions that cannot be tested with the given data, it is strongly encouraged to conduct sensitivity analyses that examine how the estimated target treatment effect varies for different assumptions regarding the underlying missing data mechanism.^11,14,34^ The estimand framework appears particularly relevant for pragmatic research questions as it can consider the disruptions (intercurrent events) that commonly arise in real-world practice in a trial-specific manner. The European Medicines Agency's ICH E9(R1) addendum introduced a well-organized structure for defining estimands, with explanations of how they influence study design and statistical analysis in clinical trials.^34^ Although the detailed presentation of these approaches is beyond the scope of this article, guidance is available.^11,14,34,110^ Overall, we concur with other authors that “*pragmatic trials require an explicit and careful definition of the effect of interest […] and the collection of high-quality longitudinal data”* (p. 1391).^68^

#### 3.11.2. Examining subgroup differences

To develop “precision” (or personalized) approaches to pain treatment, more attention must be paid to the clinical meaningfulness of study estimates, including the potential for prognostic or data-driven subgrouping. In clinical trials of pain treatments, several analytical approaches have been used to further investigate the potential heterogeneity of treatment effects and to improve the interpretation of study results, including patient subgrouping determined by clinical phenotypes and biomarkers (eg, quantitative sensory testing, epidermal innervation, and neuroimaging).^23^ These methods hold the promise that the interpretation of trial results may be greatly enhanced by the identification of patients who respond more robustly to treatment or who have fewer adverse events than others. We advocate for running pragmatic trials with fewer outcomes, as discussed above, which will make large pragmatic trials more feasible. At the same time, we believe that it is important to use improved analysis methods that examine personalization based on individual patient traits or subgroup characteristics, making best use of the collected data and moving towards personalized pain care.

For many pragmatic trials, it will be challenging to include the types of patient assessments at baseline that have been used in efficacy trials to examine differences among patients in their responses to treatment. Biomarkers often require specialized procedures that are not available in clinical practice, although efforts have been made to develop methods such as bedside sensory testing.^147^ Nonetheless, it would be possible to examine various demographic and clinical phenotypes in many pragmatic trials, for example, interactions between treatment (ie, treatment vs control) and age, sex, race, BMI, pain duration, and comorbid conditions. Such analyses should be justified and prespecified in the statistical analysis plan, and if a specific confirmatory hypothesis is being tested, adjustments for multiple comparisons must be made. Often, hypotheses about interactions between patient characteristics and treatment responses occur after data analyses have begun; the results of any post hoc analyses must be clearly identified in publications as exploratory and requiring prospective replication. Although testing the statistical significance of interactions requires larger sample sizes for adequate power, this might be less of a concern in pragmatic trials than it has been for generally smaller-sized efficacy trials given their often larger sample size.^53^ Any increase in sample size that such analyses require should be acknowledged and supported by funders when there is compelling scientific justification.

#### 3.11.3. Associations between outcomes, and benefit–risk evaluation

To guide clinical and policy decisions, it may also be necessary to evaluate associations between outcomes, for example, studying benefits and risks within individual patients, which can then allow ranking of the desirability of different outcome combinations.^35,36,39,75^ In pain research, there is the potential for a confounding effect from underlying pain-generating conditions, such as pain improving not because of better pain management but because of remission of the underlying disease (making randomized trials essential). Conversely, (un)successful pain management may affect the underlying disease outcome, as in the discontinuation of chemotherapy due to insufficient pain relief. Again, interpretation of one outcome (eg, pain relief) can require clinical context with another outcome for the same patient (eg, disease parameters or behavioral parameters). Finally, the concept of “risk” may also encompass less serious and often common adverse effects that nonetheless limit the real-world acceptability of analgesic treatments.^44,87^ All these factors are trial specific and require consideration when determining the outcomes of interest, combinations of outcomes, and analytical approach.

In our recent review, none of the included studies employed composite metrics or analyses that directly juxtaposed treatment risks and benefits.^71^ Like cost-savings, risk–benefit considerations can be implicit in some trials, namely, when a treatment is of obvious low risk. Where this is not the case, however, most authors resort to simply reporting adverse events, comparing them between groups or informally considering risk vs benefit in the trial report.^71^ However, such reporting does not allow for informed decision making in clinical practice or policy, in particular when rates of effectiveness or adverse events are similar, nor does this allow for the identification of subgroups with different risk–benefit profiles.^36^ Consider, for example, a trial of 3 interventions showing different benefit–risks profiles: Treatment A has a 50% success rate and safety events (or important side effects) occur in 30% of cases, treatments B and C both have a 50% success rate and safety events occur in 50% of cases. Based on this information, one would select treatment A, and treatments B and C are indistinguishable. However, rather than using the entire patient population to analyze 2 distinct outcomes, we can use individual patient profiles to analyze the association between outcomes. Patients may fall in one of 4 categories: (1) treatment success with a safety event, (2) success without a safety event, (3) treatment failure and a safety event occurred, and (4) failure and no safety event. If we were to find that in treatment B, success and safety events are highly correlated (ie, many patients with treatment success also had adverse events), while in treatment C, they were not, the interpretation of the trial would shift dramatically. As above, combinations of outcomes and respective analyses can answer pragmatic, clinically relevant questions. We believe such approaches to be promising especially for pragmatic trials and underused in pain research. Guidance on specific methods is available,^37,39,40^ including from IMMPACT,^98^ and composite measures have been developed.^93^

##### 3.11.3.1. Reporting

The present set of IMMPACT considerations share a unifying theme: achieving a balance between internal trial validity and obtaining meaningful information relevant to everyday clinical practice. Researchers will inevitably weigh these demands differently and must tailor methods for each research question and trial context. To comprehensively understand a trial, its report or supplementary files must elucidate the reasoning behind methodological choices, clarifying the specific pragmatic question and why the trial design is deemed suitable to address it. The CONSORT extension for pragmatic trials contains some items to that effect,^191^ but additional considerations are deemed relevant (Table 3). Furthermore, authors are encouraged to provide a self-assessment and justification of trial methods according to the PRECIS-2 domains.^111^ Note that the use of the PRECIS-2 *table*, not wheel-diagram, is preferable because it allows for a presentation of design rationales (available at https://www.precis-2.org/Help/Documentation/Toolkit).

**Table 3:** Reporting recommendations for pragmatic trials in pain research.

Recommendation for the reporting of pragmatic trials
Clearly present the pragmatic study objective, its rationale and justification, and the hypotheses that will be tested. (See Introduction section, the associated IMMPACT statement on pragmatic analgesia trials,^73^ and the CONSORT explanations and elaborations document.^122^)
Fully adhere to the current CONSORT reporting statement.^122^
Fully comply with the CONSORT extension for pragmatic trials.^191^
Provide all information necessary for the retrospective assessment of the trial by means of the PRECIS-2 instrument^111^ or present a self-assessment.
Outline how the trial methods are thought to enable answering of the pragmatic research question. (See Introduction section and the associated IMMPACT statement.^73^)
Provide information of the permissiveness and nature of concomitant and rescue medication for each group and their probable influence on trial results. (See 3.6. Concomitant and rescue pain treatments and Table 1.)
Report considerations of the relationship between benefits and risks, using statistical methods if appropriate. (See 3.11. Analysis.)
Discuss economic implications or justify why this has not been considered. (See 3.11. Analysis)
Justify the choice of the analysis population (more specifically, the estimand) in relation to the research question. (See 3.11. Analysis.)
Acknowledge the origin, reason, and potential implications of missing data. (See 3.11. Analysis.)

Considering available reporting guidelines, expanding ACTTION recommendations for efficacy trials reporting*^52^* and translating IMMPACT considerations into reporting requirements.

Box 1. **Resources for pragmatic trial design, conduct, reporting, and interpretation.**

(1) Theme issue on pragmatic and virtual trials in the journal Contemporary Clinical Trials (Volumes 113-119): https://www.sciencedirect.com/journal/contemporary-clinical-trials/special-issue/10P5MQC6F0V
(2) Article series: Pragmatic trials and real world evidence in the Journal of Clinical Epidemiology (Volumes 88-91): https://www.sciencedirect.com/journal/journal-of-clinical-epidemiology/special-issue/10X9857N20G
(3) NIH Collaboratory Rethinking Clinical Trials—The Living Textbook: https://rethinkingclinicaltrials.org/
(4) CONSORT extension for pragmatic trials^191^
(5) PRECIS-2 tool^111^ and website: https://www.precis-2.org/

Box 2. **Take home messages.**

(1) Trial designers must ensure that a trial can reliably answer the study question.
(2) In pragmatic trials, doing so requires balancing the requirements of internal and external validity, providing generalizable but reliable results.
(3) Feasibility of trials must also be considered but does not justify design choices that result in uninterpretable or low-quality studies.
(4) In pain research, specific considerations include
  (a) Selecting patient-centered meaningful outcomes, assessing the feasibility of data collection, possibly optimizing data collection from electronic health records, and exploring ultra-brief measures and diverse primary outcomes beyond pain intensity.
  (b) Exploring remission criteria, changes in medication, healthcare utilization, and treatment adherence as objective indicators of therapeutic success.
  (c) Employing analysis models that precisely define the effect of interest and account for disruptions in care common in real-world pain practice.
  (d) Advancing personalized care, eg, with adaptive trial designs, consideration of patient subgroups, individualized outcomes, and associations between beneficial and undesirable treatment effects.
(5) All pragmatic analgesia trials can be improved by
  (a) Rigorously implementing basic methods to enhance internal validity (eg, blinding of outcome assessors and randomization).
  (b) Preparing trials by considering the influence of any design choice on internal validity, generalizability, and trial feasibility.
  (c) Considering the potential of novel technological and statistical approaches.
  (d) Improving reporting.

In reporting a trial intended for clinicians and decision makers, authors should write the report with these end users in mind: Trial authors are ideally positioned to enable others to judge the applicability of findings to their setting and population, and authors should provide essential information, currently frequently omitted or superficially reported.^71^ Details, such as who was treated, how, by whom, and in which context, are crucial for end users of pragmatic trials and warrant detailed reporting. Word limit policies may hinder comprehensive reporting, and editors should consider permitting extensive online supplementary material for authors to offer detailed descriptions of interventions, trial populations, settings, and procedures. Finally, trial reports should facilitate evidence synthesis and meta-research.^70^ For any trial, this includes detailed reporting of participant numbers at each time point as well as outcome data per group,^109,156^ and ideally making individual participant data openly available. The International Committee of Medical Journal Editors (ICMJE) guidelines also encourage separate reporting of data by demographic variables.^81^ When involving pragmatic trials, systematic reviews benefit from information to judge the applicability of trial results,^191^ and meta-analyses by being able to incorporate information about within-study heterogeneity to evaluate the appropriateness of data pooling^120^ or to explain variations in effect sizes.^153^

Specific reporting recommendations are proposed in Table 3.

## 4. Discussion

For years, researchers and regulatory bodies have called for improved pragmatic and comparative effectiveness trials. In 2009, Luce et al^112^ called for the research community to focus more on the comparison of multiple interventions in routine practice settings and on designs that make it possible to funnel resources into the most promising approaches while allowing for adaptation in the light of emerging evidence. They highlight the need to efficiently collect data, use all data collected during a trial, and embrace subgroup analyses of treatment responses.^112^ At the same time, and like many other authors,^48,167^ Luce et al.^112^ draw attention to the basic challenge of pragmatic trials: If a trial fails to show effectiveness for a promising intervention, how can we know if this was because the treatment really is not effective in a broader population and real-world setting—or if it was due to factors inherent in the design and conduct of the trial? This balancing of external and internal validity is the basic theme of the present article series.^73^ Our work is aligned with a recent topical review by Keefe et al.,^94^ which made related recommendations for embedded and implementation trials of behavioral pain treatments. Here, we expand these considerations to analgesic trials in general and add further topics. This article adds to existing recommendations^69,111,187^ by making pain-specific recommendations where required. It also emphasizes the need for a deliberate consideration of the tension between internal and external validity in each design decision. With some exceptions,^51,56,57,187^ prominent guidance to date focuses on enhancing clinical applicability.^77,111,140,172^

Trial designers need to ensure that their study can answer the question under investigation and do so in a rigorous manner. We acknowledge that fully complying with all recommendations holds the risk of pragmatic trials becoming overly large and complicated or losing their resemblance to routine practice.^148^ On the other hand, uninterpretable trials add to research waste^141^; our publications can help trialists and funders to minimize such. We also hold that there is a lot to be gained from (1) deliberately weighing design choices regarding their influence on internal validity, generalizability, and trial feasibility during the planning phase, (2) performing basic methods well, such as blinding of outcome assessors and randomization, known to enhance trial validity (see Ref. 73), (3) harnessing novel technological and statistical approaches as outlined in this article, and (4) raising the standard of trial reporting. We have presented considerations specific to the field of pain research, but many are likely transferable to other fields.

## Conflict of interest statement

The first author Dr Hohenschurz-Schmidt was renumerated by IMMPACT for their work at the consensus meeting and in drafting the manuscript. The project was supported by ACTTION, a public–private partnership. The views expressed in this article are those of the authors and no official endorsement by the Food and Drug Administration (FDA) or the pharmaceutical and device companies that provided unrestricted grants to support the activities of the ACTTION public–private partnership should be inferred. Individual authors' declarations of potential conflicts of interest are as follows: Dr Bair reports grants or contracts from VA Health Services Research and Development, VA Cooperative Studies Program, and National Endowment for the Arts; participation on a Data Safety Monitoring Board or Advisory Board on a 1 NIH project conducted at the University of Utah. This trial is a pragmatic trial of physical therapy intervention; Prof Cherkin reports being paid an honorarium for mentoring the first author with manuscript writing; Prof DeBar reports support for the present manuscript from Kaiser Permanente Washington Health Research Institute (KPWHRI); grants and contracts from National Institutes of Health (NIH), and Patient Centered Outcomes Research Institute (PCORI); an honorarium for a lecture at 2020 IMMPACT Consensus Meeting; support for attending meetings from KPWHRI, and NIH; participation on a Data Safety Monitoring Board or Advisory Board for NCCIH, BACPAC DSMB; Mrs Cowan is the cofounder and secretary of the World Patients Alliance and Board Member Emeritus as well as founder of the American Chronic Pain Association; Prof Dworkin has received in the past 5 years research grants and contracts from the US Food and Drug Administration and the US National Institutes of Health, and compensation for serving on advisory boards or consulting on clinical trial methods from Abide, Acadia, Adynxx, Analgesic Solutions, Aptinyx, Aquinox, Asahi Kasei, Astellas, Beckley, Biogen, Biohaven, Biosplice, Boston Scientific, Braeburn, Cardialen, Celgene, Centrexion, Chiesi, Chromocell, Clexio, Collegium, CoimbiGene, Concert, Confo, Decibel, Editas, Eli Lilly, Endo, Ethismos (equity), Eupraxia, Exicure, GlaxoSmithKline, Glenmark, Gloriana, Grace, Hope, Hospital for Special Surgery, Lotus, Mainstay, Merck, Mind Medicine (also equity), Neumentum, Neurana, NeuroBo, Novaremed, Novartis, OCT, Orion, OliPass, Pfizer, Q-State, Reckitt Benckiser, Regenacy (also equity), Sangamo, Sanifit, Scilex, Semnur, SIMR Biotech, Sinfonia, SK Biopharmaceuticals, Sollis, SPRIM, Teva, Theranexus, Toray, Vertex, Vizuri, and WCG; Prof Edwards reports no conflicts of interest; Prof Evans reports consulting fees from advantagene, AstraZeneca, AtriCure, Degruyter, FHI clinical, Genentech, Horizon Pharma plc, International Drug Development Institute, lung biotech, microbiotix, Neovasc Medical Inc, nobel pharma, roivant, and sab pharma; support for attending meetings or travel from ACTTION, Antimicrobial Resistance and Stewardship Conference, Clinical Trials Transformation Initiative, Council for International Organizations of Medical Sciences, Deming Conference, and the FDA; participation on a Data Safety Monitoring Board or Advisory Board for AbbVie, advantagene, Akouos, Alexion Pharmaceuticals, Inc., Apellis, Breast international group, clover, DayOneBio, Duke Clinical Research Institute, FHI clinical, lung biotech, the NIH, nuvelution, Perelman School of Medicine, University of Pennsylvania, Pfizer, Rakuten, Roche, sab pharma, Takeda Oncology, Teva Pharmaceuticals Industries, tracon, and vir; and other royalties from or interests in the Clinical Trials Transformation Initiative, Degruyter, Deming Conference, the FDA, and Taylor & Francis; Prof Farrar reports grants or contracts from the NIH-NCATS – UL1 Grant (Co-I), FDA-BAA Contract, NIH-NIDDK - U01 Grant (CoI), and NIH-NINDS—U24 Grant (PI); consulting fees from Lilly and Vertex Pharma; Participation on a Data Safety Monitoring Board or Advisory Board for NIH-NIA(DSMB); and a role as President Elect US-ASP; Dr Ferguson reports grants or contracts from ACTTION paid to her institution for work on systematic reviews and payment or honoraria for lectures at IMMPACT meetings from ACTTION; Prof Freeman reports consulting fees from AlgoRx, Allergan, Applied Therapeutics, Clexio, Collegium, Cutaneous NeuroDiagnostics, Glenmark, GW Pharma, Glaxo-Smith Kline, Eli Lilly, Lundbeck, Maxona, Novartis, NeuroBo, Regenacy, Vertex, and Worwag; and stock options in Cutaneous Neurodiagnostic Life Sciences, NeuroBo, Maxona, and Regenacy; Dr Gewandter reports grants or contracts from the NIH; consulting fees from AlgoTX, GW Pharma, Magnolia Nuerosciences, Orthogonal, Science Branding Consulting, AKP Pharma, and Eikonizo; and support for attending meetings or travel from SOPATE and INS; Prof Gilron declares a travel stipend to attend ACTTION meeting 2019 and reports consulting fees from Combigene, GW Research, Lilly, and Novaremed; Prof Grol-Prokopczyk reports grants or contracts from the National Institute on Aging of the National Institutes of Health, Award #R01AG065351; and honoraria for an invited lecture for Multidisciplinary Research in Gerontology Colloquium Series, University of Southern California and for an invited lecture at Napa Pain Conference; and travel reimbursement for travel to Napa Pain Conference; Dr Hohenschurz-Schmidt reports support for the present manuscript from a PhD Studentship by the Alan and Sheila Diamond Charitable Trust and a honorarium from IMMPACT; a research grant from The Osteopathic Foundation (paid to institution); consulting fees from Altern Health Ltd.; and the role of executive committee member of the Society for Back Pain Research; Prof Iyengar reports employment and travel support from the NINDS/NIH; stock options in Retiree, and Eli Lilly and Company; other financial or nonfinancial interests through Employee of NINDS/NIH; Adjunct Senior Research Professor, Indiana University School of Medicine, Departments of Anesthesia and Clinical Pharmacology; Prof Kamp reports support for the present manuscript from ACTTION, FDA contract #HHSF223201000078C, with payments made directly to her institution, University of Rochester Medical Center, providing 5% salary support, consulting fees from Clintrex Research Corporation (payments made to her consulting company CLKamp Consulting LLC with none of the consulting in relation to the indication of pain); Dr Karp declares no conflict of interest; Prof Kerns reports Honoraria for presentation at the IMMPACT consensus meeting that informed this manuscript; research grants from NIH, PCORI, and VA paid to his institution; a consulting fee for a NIH-sponsored research grant; a honorarium for planning and participation in an IMMPACT consensus conference on Patient Engagement in Clinical Pain Research; honoraria for participation in NIH and PCORI DSMBs and a honorarium for participation as member of Scientific Advisory Board, Chronic Pain Centre of Excellence for Canadian Veterans; an unpaid role on the Board of Directors, A Place to Nourish your Health; and an honorarium for role as Executive Editor, *Pain Medicine*; Dr Kleykamp reports income from ACTTION as a full-time employee Oct 2018 - Aug 2021; being the owner and principal of BAK and Associates, LLC a research and science writing consulting firm. Contracts to BAK and Associates, LLC over the last 36 months include: STATinMED, American Society of Addiction Medicine, ECRI, Hayes/Symplr, PinneyAssociates, and Palladian Associates; payments and honoraria for lectures, presentations, speakers bureaus, manuscript writing or educational events from University of Kentucky, STATinMED, Filter Magazine, and Virginia Commonwealth University; support for attending meetings or travel from University of Kentucky and Virginia Commonwealth University; and a role as Communications Chair for the College on Problems of Drug Dependence; Prof Loeser reports no conflict of interest; Prof Mackey reports support for the present manuscript from the National Institutes of Health, US Food and Drug Administration, Patient-Centered Outcomes Research Institute, Chris Redlich Professorship in Pain Research, and Dodie and John Rosekrans Pain Research Endowment Fund (all through the Stanford University); consulting fees from Oklahoma University – Smith NIH Grant; payments or honoraria for lectures, presentations, speakers bureaus, manuscript writing or educational events Memorial Sloan, Kettering Cancer Center, National Institutes of Health, Washington University, Oakstone Publishing, Comprehensive, Review of Pain Medicine CME Lecture Series, Walter Reed AFB, Web Based Lecture, Bull Publishing, George Washington University, University of Washington, Veterans Affairs, HSRD Naloxone Distribution IIR Advisory Board, Canadian Pain Society, National Institutes of Health, and New York University; support for attending meetings or travel from American Academy of Pain Medicine, American Society of Regional Anesthesia and Pain Medicine, Washington University, George Washington University, National Institutes of Health, University of Washington, US Federal Drug Administration, New York University, Weill Cornell Medical College, and the International Neuromodulation Society (INS); roles on the Drug Safety and Risk Management Advisory Committee, Anesthetic and Analgesic Drug Products Advisory Committee (DSaRM/AADPAC)/(FDA) (Unpaid role as Advisory Committee Member) and for HSRD Naloxone Distribution IIR Veterans Affairs (VA) (honorarium paid to himself for role as Advisory Board Member); an unpaid role as Vice-Chair - Committee on Temporomandibular Disorders for the National Academies of Sciences, National Institutes of Health, (NAS)/(NIH); and other financial interests through the National Institutes of Health for T32 Postdoctoral Fellows who conduct research in lab; Salary supported by NIH and administered through Stanford University; Dr Malamut reports no conflicts of interest; Prof McDermott declares grants or contracts from the NIH, US Food and Drug Administration, Cure SMA, and PTC Therapeutics; consulting fees from Fulcrum Therapeutics, Inc. and NeuroDerm, Ltd.; Participation on a Data and Safety Monitoring Board or Advisory Board for the NIH, Eli Lilly and Company, Catabasis Pharmaceuticals, Inc., Vaccinex, Inc., Neurocrine Biosciences, Inc., Voyager Therapeutics, Prilenia Therapeutics Development, Ltd., ReveraGen BioPharma, Inc., and NS Pharma, Inc.; Prof McNicol reports grants or contracts from ACTTION paid to his institution for work on systematic reviews and payment or honoraria for lectures and attending IMMPACT meetings from ACTTION; Prof Patel reports grants and contracts from the US Centers for Disease Control and Prevention and the National Institutes of Health and consultancy work for GlaxoSmithKline LLC; Prof Rice reports support for the present manuscript from IMMPACT; grants and studentships from UKRI (Medical Research Council and BBSRC), Versus Arthritis, Royal British Legion, European Commission, UK Ministry of Defence, Dr Jennie Gwynn Bequests, Alan and Sheila Diamond Trust, the British Pain Society, and the Royal Society of Medicine; consultancy and advisory board work for Imperial College Consultants, which, in the last 36 months, has included remunerated work for: Confo, Vertex, Pharmanovo, Lateral, Novartis, Mundipharma, Orion, Shanghai SIMR BiotechAsahi Kasei & Toray; lecture honoraria from MD Anderson Cancer Center, Royal Marsden Hospital, and Ucsf; Prof Rice is named as an inventor on patents: Rice A.S.C., Vandevoorde S. and Lambert D.M Methods using N-(2-propenyl)hexadecanamide and related amides to relieve pain. WO 2005/079771 & Okuse K. et al. Methods of treating pain by inhibition of vgf activity EP13702262.0/WO2013 110945; a role as Chair of the Trial Steering Committee (TSC) for the OPTION-DM trial, National Institute for Health Research (NIHR); role as councilor for IASP and current position as president-elect; he also was the owner of share options in Spinifex Pharmaceuticals from which personal benefit accrued upon the acquisition of Spinifex by Novartis in July 2015. The final payment was made in 2019; other interests are in the British National Formulary, Joint Committee on Vaccine and Immunisation-varicella sub-committee, Medicines and Healthcare products Regulatory Agency (MHRA), Commission on Human Medicines - Neurology, Pain & Psychiatry Expert Advisory Group, Non Freezing Cold Injury Independent Senior Advisory Committee (NISAC), and Royal College of Anaesthetists - Heritage and Archives Committee; Prof Rowbotham reports consulting fees from SiteOne Therapeutics, GenEdit and Sustained Therapeutics, payments for expert testimony from Haapala, Thompson & Abern (law firm for clinical payment to himself as medical-legal expert witness), payments from Helixmith Co., LTD for work on a data monitoring or advisory board, and unpaid work as Treasurer of the International Association for the Study of Pain from 2020-2024. He also holds stock options from SiteOne and CODA Biotherapeutics; Dr Schmader reports a grant by GSK for vaccine research, paid to his institution; Dr Steiner reports being full-time employee of Eli Lilly and Company (Pain & Neurodegeneration); Dr Simon reports consulting fees from Astrazeneca, Pfizer, Rigel, Eupraxia, Biosplice, EMDSerono, Horizon, Direct, Lilly, Kaniska, Protalix, Chemomab, TLC, SpineThera, Kyoto, PPD, Galvani, Urica, Transcode, Boehringer Ingelheim, Bristol Myers Squibb, Priovant, Roivant, Ampio, Aura, Aurinia, GSK, Xalud, Neumentum, Neema, Amzell, Applied Bio, Aptinyx, Bexson, Bone Med, Bone Therapeutics, Cancer Prevention, Cerebral Therapeutics, Chemocentryx, Diffusion Bio, Elorac, Enalare, Foundry Therapeutics, Galapagos. Histogen, Gilead, Idera, Intravital, Ingel, Kiel Labs, Mesoblast, Mpathix, Minerva, Regenosine, Samus, Sana, StageBio, Theraly, Unity, and Viridian; Prof Turk reports royalities and licenses from Wolters Kluwer (Editor-n-Chief, Clinical Journal of Pain), and the American Psychological Association (Book Author); consulting fees from GSK/Novartis; and a role as Associate Director of Analgesic, Anesthetic, and Addiction Clinical Trials, Innovations, Opportunities and Networks (ACTTION); Dr Veasley reports no conflicts of interest; Dr Vollert reports consulting fees from Vertex Pharmaceuticals, Embody Orthopaedic, and Casquar. Finally, Prof Wasan reports no conflicts of interest.
